# From obligation to action: unraveling the roles of social responsibility and prosocial tendency in shaping Chinese doctors’ vaccine hesitancy

**DOI:** 10.3389/fpsyt.2024.1462073

**Published:** 2024-10-02

**Authors:** Xikun Li, Yuwei Zhang, Xinyang Li, Botang Guo

**Affiliations:** ^1^ Department of Public Health, Harbin Traditional Chinese Medicine Hospital, Harbin, Heilongjiang, China; ^2^ College of Art Academy, Northeast Agricultural University, Harbin, Heilongjiang, China; ^3^ College of Liberal Arts, Heilongjiang University, Harbin, Heilongjiang, China; ^4^ Department of Medical Psychology, Harbin Medical University, Harbin, Heilongjiang, China; ^5^ Department of General Practice, The Affiliated Luohu Hospital of Shenzhen University Medical School, Shenzhen, China

**Keywords:** vaccine hesitancy, social responsibility, prosocial tendency, Chinese doctors, mediation

## Abstract

**Background:**

The hesitation of healthcare professionals towards vaccines is becoming increasingly concerning, potentially undermining public confidence in vaccination programs. This study aimed to investigate the relationship between social responsibility, Prosocial tendency, and vaccine hesitancy among Chinese doctors, and to identify demographic factors associated with vaccine hesitancy.

**Method:**

A cross-sectional survey was conducted among 976 Chinese doctors. Participants completed a questionnaire to assess their sense of social responsibility, Prosocial tendency, and vaccine hesitancy. Demographic information, including age, gender, and marital status, was also collected. Correlation and mediation analyses were conducted to examine the relationships between the main variables.

**Results:**

Social responsibility was negatively correlated with vaccine hesitancy (*r*=-0.564, *P*<0.01) and positively correlated with Prosocial tendency (*r*=0.519, *P*<0.01). Prosocial tendency was negatively correlated with vaccine hesitancy (*r*=-0.505, *P*<0.01) and partially mediated the relationship between social responsibility and vaccine hesitancy. Younger age, female gender, and unmarried status were associated with higher levels of vaccine hesitancy.

**Conclusion:**

This study emphasized the important role of social responsibility and Prosocial tendency in reducing vaccine hesitancy among Chinese doctors. The findings suggest that interventions aimed at fostering a strong sense of social responsibility and promoting Prosocial tendency may effectively address vaccine hesitancy in this population. Additionally, targeted interventions focusing on young, female, and unmarried doctors may be necessary.

## 1 Introduction

Vaccine hesitancy, defined as the delay in acceptance or refusal of vaccination despite availability of vaccination services (1), has become a growing challenge for immunization programs globally. While much research has focused on vaccine hesitancy among the general public, the issue of vaccine hesitancy among healthcare workers, particularly doctors, is also concerning (2, 3). In China, vaccine hesitancy among doctors has emerged as a significant problem in recent years. A meta-analysis by Wang et al. (4) found that the influenza vaccination coverage among healthcare workers in China was only 17.7%, suggesting a high level of vaccine hesitancy in this population. Similarly, a national cross-sectional study by Fu et al. (5) reported that 23.4% of healthcare workers in China intended to accept COVID-19 vaccination. Li et al. (6) reported that healthcare workers had positive attitudes towards future COVID-19 vaccines, while vaccine hesitancy was still common. The high prevalence of vaccine hesitancy among doctors is a serious public health concern (7). As trusted sources of medical information, doctors’ personal attitudes and practices towards vaccines can have a significant impact on their patients’ willingness to accept and receive vaccinations (8). Vaccine-hesitant doctors may be less likely to provide strong recommendations for vaccines or to effectively address patients’ concerns regarding vaccine safety and effectiveness (9, 10).

Moreover, vaccine hesitancy among doctors can have a ripple effect on the wider community. As respected members of society, doctors’ vaccine attitudes and behaviors can shape social norms and influence public opinion on vaccination (11). If doctors are seen to be questioning the value of vaccines, it can fuel doubts and misconceptions among the general public, leading to decreased vaccine confidence and uptake. The problem of vaccine hesitancy among Chinese doctors is compounded by the fact that China has a large and diverse population, with varying levels of education, socioeconomic status, and access to healthcare (12). In this context, doctors play an even more critical role in building trust in vaccines and ensuring equitable access to immunization services. Therefore, it is crucial to understand the factors influencing vaccine hesitancy among doctors in order to develop targeted interventions to address this issue.

Vaccine hesitancy is a complex and multidimensional concept that encompasses an individual’s attitudes, beliefs, and behaviors towards vaccination (1). Under the framework of Planned Behavior Theory (TPB), vaccine hesitancy can be understood as a comprehensive reflection of an individual’s attitude towards vaccination (such as concerns about vaccine safety and efficacy), subjective norms (such as perceived social pressure), and perceived behavioral control (such as barriers to obtaining vaccines) (13). These factors collectively affect an individual’s willingness to receive vaccinations, which in turn affects their actual vaccination behavior. Previous research has identified various factors associated with vaccine hesitancy, including knowledge, attitudes, and beliefs about vaccines and vaccine-preventable diseases (14). One potential factor that has received less attention is social responsibility, which refers to an individual’s sense of duty and obligation to act in the best interests of society (15). According to the theory of planned behavior, an individual’s behavior is determined by their intentions, which are influenced by their attitudes, subjective norms, and perceived behavioral control (16). In the context of vaccination, a doctor’s sense of social responsibility may shape their attitudes towards vaccines and their perceived moral obligation to get vaccinated to protect others.

Similarly, social norms theory posits that individuals’ behaviors are heavily influenced by their perceptions of what is socially acceptable or expected within their community (17). Doctors who perceive a strong social norm of responsibility and altruism within the medical community may feel more compelled to get vaccinated as part of their professional duty.

Empirical studies have provided support for the link between social responsibility and vaccine attitudes and behaviors. For example, a study by Betsch and Böhm (18) found that individuals with higher levels of prosocial orientation, which is closely related to social responsibility, were more likely to get vaccinated against influenza. Another study by Shim et al. (19) showed that a sense of social responsibility was a significant predictor of college students’ intentions to receive the H1N1 vaccine.

In the context of healthcare workers, research has shown that a strong sense of professional responsibility is associated with more positive attitudes towards vaccination. A study researched by Yu et al. (20) found that medical college students in China who felt a greater sense of social responsibility were more likely to accept vaccines for themselves.

Moreover, studies have suggested that interventions designed to enhance healthcare workers’ sense of social responsibility can effectively reduce vaccine hesitancy. For instance, a randomized controlled trial by Shelby and Ernst (21) demonstrated that a brief intervention emphasizing nurses’ professional responsibility to protect patients led to a significant increase in their intentions to receive the influenza vaccine. Based on this theoretical and empirical evidence, we hypothesize that doctors’ sense of social responsibility will be negatively associated with their vaccine hesitancy.

Furthermore, we propose that prosocial tendency may play a crucial role in the relationship between social responsibility and vaccine hesitancy. Prosocial tendencies referred to an individual’s dispositional inclination to engage in actions that benefit others (22). Prosocial tendencies are characterized by empathy, concern for others’ welfare, and the motivation to help and support those in need. The link between social responsibility and prosocial tendencies can be understood through the lens of social identity theory (23). These theories posit that individuals who strongly identify with a social group or category (e.g., society as a whole) are more likely to internalize the norms, values, and goals associated with that group. Consequently, they are more motivated to engage in behaviors that align with the group’s interests and contribute to its well-being.

In the context of our study, healthcare workers with a strong sense of social responsibility are likely to view themselves as part of a larger social collective and feel a moral obligation to act in ways that benefit society. This heightened sense of social responsibility may activate prosocial tendencies, as healthcare workers become more attuned to the needs of others and are motivated to engage in actions that promote the well-being of their patients, colleagues, and the broader community. Empirical evidence supports the positive relationship between social responsibility and prosocial tendencies. Studies have shown that individuals with higher levels of social responsibility are more likely to engage in charitable giving (24), and environmentally responsible behaviors (25). These findings suggest that a strong sense of social responsibility can indeed foster prosocial tendencies and motivate individuals to act in ways that benefit others and society as a whole.

Prosocial tendency is defined as voluntary actions intended to help or benefit others, often at a personal cost to the individual performing the action (26). This concept is closely related to social responsibility, as individuals with a strong sense of duty and obligation to society may be more inclined to engage in prosocial acts (27).

The link between Prosocial tendency and vaccine attitudes can be understood through the lens of the theory of planned behavior. According to this theory, an individual’s behavior is influenced by their attitudes, subjective norms, and perceived behavioral control, which collectively shape their intentions to perform the behavior (16). In the context of vaccination, doctors who view getting vaccinated as a prosocial act that benefits others may have more positive attitudes towards vaccines and stronger intentions to get vaccinated. Empirical research has provided support for the association between prosocial tendencies and vaccine acceptance (28). For example, a study by Böhm et al. (29) found that individuals with higher levels of prosocial orientation were more likely to get vaccinated against influenza, even when the vaccine was less effective in protecting the individual. Also, several experimental studies found that prosociality was positively associated with preventive behaviors against COVID-19 (e.g., social distancing and facemask wearing) (30–32).

Moreover, Prosocial tendency may mediate the relationship between social responsibility and vaccine hesitancy. Doctors with a strong sense of social responsibility may be more likely to engage in prosocial acts, such as getting vaccinated, to protect their patients and community. This increased Prosocial tendency, in turn, may lead to reduced vaccine hesitancy. The mediating role of Prosocial tendency is consistent with the theory of planned behavior, which suggests that attitudes and beliefs influence behavior through the mediating role of intentions. Based on this theoretical and empirical evidence, we hypothesize that Prosocial tendency will mediate the relationship between social responsibility and vaccine hesitancy among doctors.

### 1.1 Current study

Based on the aforementioned literature review and theoretical framework, this study aims to explore the relationship between social responsibility, prosocial tendencies, and vaccine hesitancy. We propose the following research hypothesis:

H1: Social responsibility is negatively correlated with individual vaccine hesitancy.

H2: Prosocial tendencies mediate between social responsibility and vaccine hesitancy. Specifically, the stronger the sense of social responsibility, the higher the individual’s pro social inclination, which in turn leads to a decrease in vaccine hesitancy.

**Figure 1:**
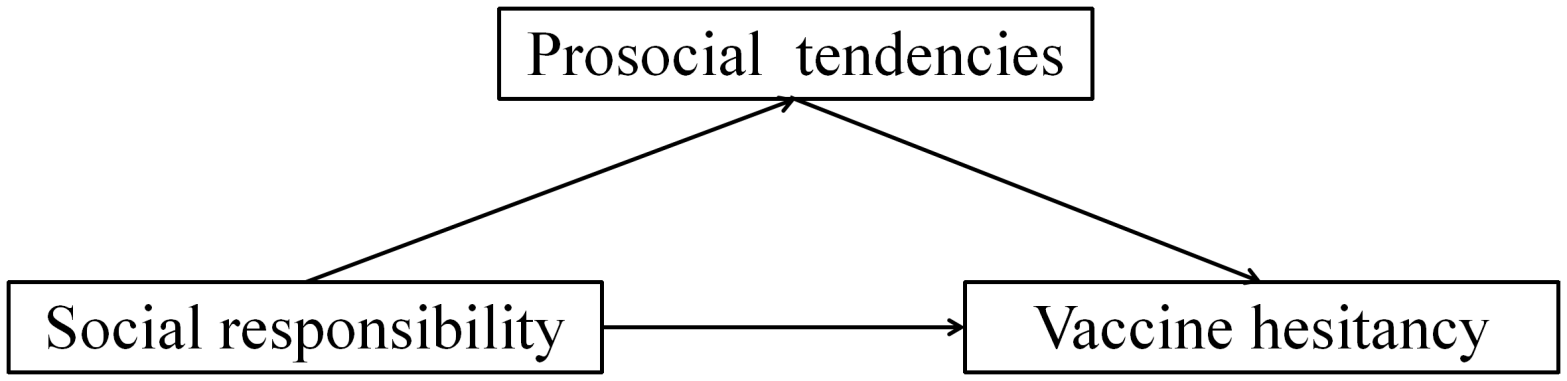
The hypothetical conceptual model.

To test these hypotheses, we constructed a mediation model (**Figure 1**). Under the framework of Planned Behavior Theory, we position social responsibility as subjective norms or attitudes, pro social tendencies as behavioral intentions, and vaccine hesitancy as a comprehensive reflection of attitudes, subjective norms, and perceived behavioral control. The contribution of this study lies in: firstly, we explored the relationship between social responsibility, a new influencing factor, and vaccine hesitancy, expanding the existing research perspective on the influencing factors of vaccine hesitancy; Secondly, we have revealed the mediating mechanism of pro social bias in the impact of social responsibility on vaccine hesitancy, providing new insights into the formation mechanism of vaccine hesitancy; Thirdly, we applied TPB to vaccine hesitancy research, providing new empirical support for the application of this theory in the field of health behavior.

## 2 Methods

### 2.1 Study design and participants

A nationwide cross-sectional online survey was conducted among Chinese doctors from January to March 2024. The inclusion criteria for participants were: (1) currently practicing as a licensed doctor in China; (2) willing to participate in the study and provide informed consent; (3) able to understand and complete the online questionnaire independently. The exclusion criteria were: (1) retired or non-practicing doctors; (2) incomplete questionnaires with missing data on key variables; (3) response time less than 5 minutes or greater than 60 minutes, indicating potentially invalid responses. This study was approved the Ethics Committee of the Harbin Traditional Chinese Medicine Hospital (HRBTCMH20240301).

### 2.2 Sample size calculation

The sample size was calculated using the formula for cross-sectional studies: n= Z²p(1-p)/d², where Z is the Z-value for the desired confidence level (1.96 for 95% CI), p is the expected proportion of doctor with influenza vaccine hesitancy (estimated as 0.8 based on previous studies (5, 6)), and d is the desired precision (set at 0.05). After calculation, 289 participants are needed, and considering a 20% non-response rate, the final sample size was determined to be 347. During the data collection process, we distributed a total of 1000 questionnaires and collected 988 responses. All participants met the inclusion criteria and were not excluded. During the data cleaning process, we excluded 16 questionnaires with poor data quality (such as a large number of missing values, a single answering pattern, etc.) or repeated responses. In the end, we obtained 972 valid questionnaires with an effective recovery rate of 97.2%.

### 2.3 Measurements

A self-administered online questionnaire was used to collect data from Chinese doctors. The questionnaire consisted of the following sections:(1)Demographic information Participants’ demographic characteristics were obtained, including age, gender, place of residence, marital status, education level, professional title, years of practice, monthly income, and department.(2)Social responsibility The 28-item Social responsibility Scale developed by Tian et al. (33) was used to assess participants’ sense of social responsibility from three dimensions: country, collective, and family(“As a family member, I have a responsibility to maintain the stability of the family. “ & “I believe that realizing the Chinese Dream is our mission.”). Each item was rated on a 5-point Likert scale ranging from 1 (completely disagree) to 5 (completely agree). Higher total scores indicated a stronger sense of social responsibility. The Cronbach’s α coefficient for this scale in the current study was 0.88. We conducted confirmatory factor analysis on the scale and the results showed that the questionnaire had good structural validity. *χ* ^2^/*df*=2.58, CFI=0.92, TLI=0.90, RMSEA=0.04. (3)Prosocial tendency Prosocial tendency was measured using the Prosocial Tendencies Measure adapted by Cong et al. (34) based on the original scale developed by Carlo and Randall (22). The scale consists of 23 items assessing prosocial tendencies across six dimensions: altruism, compliance, emotional, anonymous, public, and dire(“I prefer anonymous donations.” & “I am more inclined to help those who are severely injured.”). Responses were given on a 5-point Likert scale ranging from 1 (completely disagree) to 5 (completely agree), with higher total scores reflecting more frequent engagement in Prosocial tendencys. The Cronbach’s α coefficient for this scale in the present study was 0.92. We conducted confirmatory factor analysis on the scale and the results showed that the questionnaire had good structural validity. *χ* ^2^/*df*=1.940,CFI=0.958, TLI=0.947, RMSEA=0.039. (4)Vaccine hesitancy A 10-item questionnaire was used to evaluate participants’ level of vaccine hesitancy. The questionnaire was developed based on the Vaccine Hesitancy Scale (VHS) proposed by the SAGE Working Group on Vaccine Hesitancy (14) and adapted to the Chinese context(“Vaccines are effective.” & “Getting vaccinated is important for the health of those around you.”). Each item was scored on a 5-point Likert scale, with higher total scores indicating greater vaccine hesitancy. The Cronbach’s α coefficient for the vaccine hesitancy questionnaire in this study was 0.85.We conducted confirmatory factor analysis on the scale and the results showed that the questionnaire had good structural validity. *χ* ^2^/*df*=2.529,CFI=0.996, TLI=0.994, RMSEA=0.034.

### 2.4 Collection and quality control

Quality control measures were implemented throughout the survey process. In the process of sampling and survey implementation, we value both the geographical representativeness of the samples and the operability of the survey. According to the geographical location of China, Harbin (Northern region), Guangzhou (Southern region), Chengdu (Western region), Nanjing (Eastern region), and Zhengzhou (Central region) were selected as the research object sources. In each survey city, we collaborate with local physician associations to issue research invitations to medical institutions at all levels within our jurisdiction, and solicit doctors who voluntarily participate in the survey. At the same time, we also adopted a snowball sampling method and asked the participating doctors to recommend other suitable peers as potential research subjects. The survey is mainly conducted in the form of online questionnaires. The Medical Association distributes questionnaire links to doctors who are willing to participate in the survey via email, and doctors can fill out the questionnaire online. The online questionnaire was designed with clear instructions and a user-friendly interface. Logic checks and mandatory fields were set to minimize missing data and inconsistent responses. Participants’ IP addresses and response times were recorded to identify and exclude duplicate or invalid submissions. Reminder emails were sent one week after the initial invitation to increase the response rate.

### 2.5 Statistical analysis

SPSS 26.0 were used for data analysis. Descriptive statistics were calculated for demographic variables and key study measures. Pearson correlation was conducted to examine the associations between social responsibility, Prosocial tendency, and vaccine hesitancy. PROCESS Model 4 was performed to test the mediating effect of Prosocial tendency on the relationship between social responsibility and vaccine hesitancy, controlling for demographic covariates. The significance of the mediation effect was determined using the bootstrap method with 5000 samples. A two-tailed *p*-value less than 0.05 was considered statistically significant.

## 3 Results

### 3.1 Descriptive statistics

A total of 976 valid questionnaires were collected, with a response rate of 97.6%. The demographic characteristics of the participants were presented in **Table 1**. The average age of the doctors was 35.81 ± 7.53 years old, with 50.92% in the 31-40 age group. There were slightly more female doctors (53.07%) than male doctors (46.93%). The majority of the participants (74.90%) were from urban areas and held an undergraduate degree (77.05%). Regarding professional title, 31.97% were junior, followed by intermediate (24.69%), unclassified (19.26%), senior (13.11%), and deputy senior (10.96%).

**Table 1:** Demographic differences in doctors’ vaccine hesitancy (N = 972).

Variable	n (%)	Vaccine hesitancy Mean ± SD	F/t	P	Social responsibility Mean ± SD	F/t	P	Prosocial tendency Mean ± SD	F/t	P
Age			16.24	<.001		7.62	0.001		5.68	0.004
≤30	277 (28.38)	35.05 ± 4.58			82.70 ± 7.24			51.48 ± 12.74		
31~40	497 (50.92)	33.43 ± 3.92			84.88 ± 7.44			54.89 ± 13.74		
>40	202 (20.70)	33.14 ± 4.51			84.62 ± 8.55			53.77 ± 13.91		
Gender			-11.15	<.001		6.37	<.001		6.15	<.001
Male	458 (46.93)	32.29 ± 3.99			85.84 ± 7.40			56.48 ± 13.86		
Female	518 (53.07)	35.19 ± 4.12			82.77 ± 7.64			51.22 ± 12.81		
Residence			0.04	0.965		-0.77	0.438		0.54	0.588
Urban	731 (74.90)	33.83 ± 4.31			84.10 ± 7.72			53.82 ± 13.71		
Rural	245 (25.10)	33.82 ± 4.32			84.54 ± 7.57			53.28 ± 13.13		
Education level			1.15	0.328		0.53	0.589		0.29	0.751
Specialties	24 (2.46)	32.54± 4.80			85.63 ± 8.17			55.75 ± 14.44		
Undergraduates	752 (77.05)	33.82 ± 4.26			84.23 ± 7.76			53.62 ± 13.48		
Masters and above	200 (20.49)	34.04 ± 4.43			83.95 ± 7.34			53.70 ± 13.83		
Work Years			1.65	0.176		2.32	0.074		1.24	0.293
≤5	355 (36.37)	33.95 ± 4.24			83.68 ± 6.96			52.87 ± 13.20		
6~10	303 (31.05)	33.89 ± 4.12			84.65 ± 7.98			54.85 ± 14.09		
11~15	152 (15.57)	34.15 ± 4.37			83.48 ± 7.53			53.79 ± 13.22		
>15	166 (17.01)	33.18 ± 4.69			85.22 ± 8.58			53.23 ± 13.65		
Professional title			0.31	0.871		1.39	0.235		1.48	0.205
Unclassified	188 (19.26)	33.88 ± 4.52			83.57 ± 7.98			54.66 ± 14.69		
Junior	312 (31.97)	33.76 ± 4.19			84.68 ± 7.93			54.63 ± 13.75		
Intermediate	241 (24.69)	34.06 ± 3.90			84.00 ± 6.81			53.24 ± 13.54		
Deputy Senior	107 (10.96)	33.62 ± 4.45			83.39 ± 7.30			52.09 ± 12.31		
Senior	128 (13.11)	33.67 ± 4.90			85.09 ± 8.38			52.13 ± 12.26		
Monthly income (yuan)			0.30	0.880		2.04	0.087		0.78	0.536
≤3000	154 (15.78)	33.79 ± 4.82			85.05 ± 7.59			53.50 ± 13.13		
3001~5000	293 (30.02)	33.71 ± 4.17			84.08 ± 7.53			54.21 ± 13.56		
5001~8000	268 (27.46)	33.94 ± 4.33			84.78 ± 8.01			54.33 ± 13.79		
8001~10000	173 (17.73)	34.05 ± 4.10			82.96 ± 7.60			52.25 ± 13.52		
>10000	88 (9.02)	33.57 ± 4.18			83.91 ± 7.27			53.15 ± 13.78		
Marital status			15.07	<.001		8.95	<.001		6.14	0.002
Unmarried	275 (28.18)	35.02 ± 4.81			82.60 ± 8.22			51.32 ± 13.19		
Married	666 (68.24)	33.37 ± 3.98			84.78 ± 7.31			54.70 ± 13.58		
Other	35 (3.59)	33.26 ± 4.51			85.97 ± 8.42			53.03 ± 14.18		
Clinical department			0.26	0.937		0.68	0.639		0.98	0.426
Internal medicine	332 (34.02)	33.86 ± 4.35			84.23 ± 7.74			53.67 ± 13.48		
Surgery	326 (33.40)	33.87 ± 4.16			84.02 ± 7.34			53.01 ± 13.05		
Gynecology	93 (9.53)	33.86 ± 4.43			85.43 ± 7.95			56.43 ± 14.66		
Pediatrics	79 (8.09)	33.33 ± 4.24			83.62 ± 8.26			54.22 ± 14.07		
Emergency	80 (8.20)	33.83 ± 4.30			83.76 ± 7.54			53.24 ± 13.89		
Other	66 (6.76)	34.05 ± 4.84			84.53 ± 8.19			53.18 ± 13.89		

As shown in **Table 1**, significant differences in vaccine hesitancy were found among different age groups (*F*=16.24, *P*<0.001), gender (*t*=-11.15, *P*<0.001), and marital status (*F*=15.07, *P*<0.001). Doctors aged ≤30 years had higher vaccine hesitancy scores (35.05 ± 4.58) compared to those aged 31-40 (33.43 ± 3.92) and >40 (33.14 ± 4.51). Female doctors reported higher vaccine hesitancy (35.19 ± 4.12) than male doctors (32.29 ± 3.99). Unmarried doctors exhibited higher vaccine hesitancy (35.02 ± 4.81) compared to married doctors (33.37 ± 3.98) and those with other marital status (33.26 ± 4.51). No significant differences were observed in vaccine hesitancy based on residence, education level, work years, professional title, monthly income, or clinical department (*P*>0.05).

### 3.2 Correlation analysis

The mean scores for social responsibility, Prosocial tendency, and vaccine hesitancy were 84.21 ± 7.68, 53.69 ± 13.56, and 33.83 ± 4.31, respectively (**Table 2**). Pearson correlation analysis (**Table 2**) revealed that social responsibility was positively correlated with Prosocial tendency (*r*=0.519, *P*<0.01) and negatively correlated with vaccine hesitancy (*r*=-0.564, *P*<0.01). Prosocial tendency was also negatively correlated with vaccine hesitancy (*r*=-0.505, *P*<0.01).

**Table 2:** Description statistics and correlation analysis of each variable.

Variables	Range (min~max)	M ± SD	1	2	3
1.Social responsibility	62~112	84.21 ± 7.68	1		
2.Prosocial tendency	30~100	53.69 ± 13.56	0.519**	1	
3.Vaccine hesitancy	19~48	33.83 ± 4.31	-0.564**	-0.505**	1

^*^ *P*<0.05, ^**^ *P*<0.01.

### 3.3 Mediation analysis

Hierarchical regression analysis was conducted to examine the mediating role of Prosocial tendency in the relationship between social responsibility and vaccine hesitancy (**Table 3**). In the first step, social responsibility significantly predicted vaccine hesitancy (*β*=-0.285, *P*<0.01). In the second step, social responsibility significantly predicted Prosocial tendency (*β*=0.881, *P*<0.01). In the third step, when both social responsibility and Prosocial tendency were included as predictors, social responsibility (*β*=-0.212, *P*<0.01) and Prosocial tendency (*β*=-0.083, *P*<0.01) significantly predicted vaccine hesitancy. The effect of social responsibility on vaccine hesitancy was reduced but still significant, indicating a partial mediation effect of Prosocial tendency.

**Table 3:** Summary of hierarchical regression analyses predicting vaccine hesitancy.

Model	Regression equation		Overall fit coefficient			Regression coefficient		
	Outcome variables	Predictor variables	R	R^2^	F	β	SE	t
Model 1	Vaccine hesitancy	social responsibility	0.564	0.318	453.523^**^	-0.316	0.015	-21.296^**^
	Prosocial tendency	social responsibility	0.519	0.270	359.301^**^	0.917	0.048	18.955^**^
	Vaccine hesitancy	social responsibility	0.616	0.380	297.545^**^	-0.232	0.017	-13.967^**^
		Prosocial tendency				-0.092	0.009	-9.844^**^

Model 1: No covariates were adjusted.

*P<0.05, **P<0.01.

The bootstrap method further confirmed the significance of the mediation effect (**Table 4**). The total effect of social responsibility on vaccine hesitancy was significant (*β*=-0.285, 95%CI: -0.313 to -0.257). The direct effect accounted for 74.4% of the total effect (*β*=-0.212, 95%CI: -0.243 to -0.181), while the indirect effect through Prosocial tendency accounted for 25.6% (*β*=-0.073, 95%CI: -0.090 to -0.056).

**Table 4:** Direct and indirect effects of social responsibility on vaccine hesitancy.

Model	Path	β	SE	95%CI	Ratio of effect values
Model1	Total effect	-0.316	0.015	[-0.345,-0.287]	
	Direct effect	-0.232	0.017	[-0.264,-0.199]	73.1%
	Indirect effect	-0.084	0.009	[-0.104,-0.067]	26.9%

### 3.4 Sensitivity analysis

To assess the robustness of the mediation effect, we conducted sensitivity analyses. First, in Model 2, we included age, gender, and marital status as covariates to examine whether the mediation effect of prosocial tendencies in the relationship between social responsibility and vaccine hesitancy remained significant after controlling for these demographic variables. Second, in Model 3, we incorporated all demographic factors (including age, gender, marital status, education level, and work tenure) as covariates to further test the robustness of the mediation effect. The results of the sensitivity analyses in **Tables 5**, **6** demonstrated that the mediation effect of prosocial tendencies in the relationship between social responsibility and vaccine hesitancy remained significant even after controlling for various demographic variables, providing evidence for the robustness of the mediation model. This finding suggests that although demographic factors may have some influence on vaccine hesitancy, prosocial tendencies, as a mediating mechanism between social responsibility and vaccine hesitancy, exhibit strong universality and stability.

**Table 5:** Sensitivity analysis of hierarchical regression analyses predicting vaccine hesitancy.

Model	Regression equation		Overall fit coefficient			Regression coefficient		
	Outcome variables	Predictor variables	R	R^2^	F	β	SE	t
Model 2	Vaccine hesitancy	social responsibility	0.624	0.390	154.911^**^	-0.285	0.014	-19.750^**^
	Prosocial tendency	social responsibility	0.528	0.279	133.281^**^	0.881	0.049	17.850^**^
	Vaccine hesitancy	social responsibility	0.662	0.439	151.539^**^	-0.212	0.016	-13.287^**^
		Prosocial tendency				-0.083	0.009	-9.201^**^
Model 3	Vaccine hesitancy	social responsibility	0.629	0.396	63.216^**^	-0.285	0.014	-19.728^**^
	Prosocial tendency	social responsibility	0.538	0.290	39.364^**^	0.884	0.049	17.951^**^
	Vaccine hesitancy	social responsibility	0.666	0.444	69.864**	-0.212	0.016	-13.242^**^
		Prosocial tendency				-0.082	0.009	-9.087^**^

Model 2: Age, Gender, Marital status were adjusted.

Model 3: Age, Gender, Marital status, Residence, Education level, Work Years, Professional title, Monthly income were adjusted.

*P<0.05, **P<0.01.

**Table 6:** Sensitivity analysis of direct and indirect effects of social responsibility on vaccine hesitancy.

Model	Path	β	SE	95%CI	Ratio of effect values
Model2	Total effect	-0.285	0.014	[-0.313,-0.257]	
	Direct effect	-0.212	0.016	[-0.243,-0.181]	74.4%
	Indirect effect	-0.073	0.009	[-0.090,-0.056]	25.6%
Model3	Total effect	-0.284	0.014	[-0.313,-0.256]	
	Direct effect	-0.212	0.016	[-0.243,-0.180]	74.4%
	Indirect effect	-0.073	0.009	[-0.090,-0.056]	25.6%

Model 2: Age, Gender, Marital status were adjusted.

Model 3: Age, Gender, Marital status, Residence, Education level, Work Years, Professional title, Monthly income were adjusted.

## 4 Discussion

This study found that the vaccine hesitancy score among Chinese doctors was 33.83 ± 4.31, which is lower than the vaccine hesitancy levels observed in doctors from other countries. For instance, a study on American doctors reported a vaccine hesitancy score of 38.24 ± 6.17 (35). Similarly, a study on Italian doctors reported a score of 36.91 ± 5.82 (36). Several factors may contribute to these differences. First, China has experienced multiple major infectious disease outbreaks in recent decades, such as SARS and H1N1 influenza, which may have heightened the awareness of the importance of vaccines among Chinese doctors (37). Second, the Chinese government has implemented strong measures to promote vaccination, such as incorporating vaccination performance into professional assessments, which could influence doctors’ attitudes and behaviors (38). Lastly, the collectivist culture in China may lead doctors to place greater emphasis on social responsibility, thereby reducing vaccine hesitancy (39).

In terms of demographic characteristics, this study found that age, gender, and marital status are significantly associated with vaccine hesitancy levels among doctors. Specifically, doctors aged 30 and below have significantly higher vaccine hesitancy scores compared to those aged 31-40 and above 40. With the sudden outbreak of the COVID-19 epidemic and the acceleration of vaccine development, not only students and patients with diseases, but also medical staff are worried about the vaccine risk, especially young doctors (40–42). Young doctors may have a longer life expectancy, so they are more concerned about the long-term side effects of vaccines. Additionally, female doctors exhibit significantly higher levels of vaccine hesitancy compared to their male counterparts, which is consistent with previous research findings (43). Female doctors may be more cautious when making vaccination decisions, possibly because they not only need to consider the impact of vaccines on their own health, but also weigh the potential impact of vaccines on their children. Finally, unmarried doctors have significantly higher vaccine hesitancy levels than married doctors. In addition to the explanation that married doctors may have a stronger sense of responsibility to protect the health of their families (44), unmarried doctors’ higher vaccine hesitancy may also be related to their concerns about vaccine risks. Unmarried doctors may be more sensitive to the long-term safety of vaccines due to planning for their future family life. To address vaccine hesitancy among these subgroups, we recommend developing and implementing targeted interventions. These strategies may include disseminating reliable, evidence-based information about vaccine safety to directly address specific concerns, designing and implementing peer education programs and model demonstration projects to build confidence in vaccines, and fostering a supportive organizational climate that encourages open dialogue and the sharing of experiences and perspectives related to vaccination. Simultaneously, risk communication efforts should be enhanced for these subgroups of physicians. Health authorities and institutions should provide timely, transparent information regarding vaccine safety to help alleviate risk-related concerns. By adopting a multi-pronged approach that combines targeted interventions, peer support, and effective risk communication, vaccine hesitancy among these physician subgroups can be mitigated.

The positive correlation between social responsibility and prosocial tendency suggested that doctors with a high sense of social responsibility were more likely to engage in prosocial actions. This may be because these doctors are more concerned about the well-being of patients and society and are willing to make extra efforts to promote public health (45). For example, they might spend more time explaining the importance of vaccines to patients or proactively communicate with patients who are hesitant about vaccination (46). Furthermore, a high sense of social responsibility may motivate doctors to lead by example, actively receiving vaccinations themselves and thus setting a positive example for their patients (47).

The negative correlations between social responsibility and vaccine hesitancy, as well as between prosocial tendency and vaccine hesitancy, suggested that doctors who exhibit high levels of social responsibility and engage in more prosocial tendencys have lower levels of vaccine hesitancy. This may be because these doctors better understand the importance of vaccines in protecting individual and public health, thereby being more supportive of vaccination (48). Additionally, through prosocial tendencys such as providing vaccine-related information and addressing patients’ concerns, doctors can directly influence patients’ vaccination decisions, thereby reducing vaccine hesitancy (7).

A novel finding of this study was that Prosocial tendency partially mediated the relationship between social responsibility and vaccine hesitancy. This result suggested that enhancing doctors’ sense of social responsibility could reduce vaccine hesitancy by promoting Prosocial tendency. According to the Theory of Planned Behavior, an individual’s behavior is determined by their behavioral intentions, which are influenced by attitudes, subjective norms, and perceived behavioral control. In this study, social responsibility could be seen as a subjective norm, where doctors felt pressure from society and their professional community to fulfill their obligation to promote public health. This subjective norm could positively influence doctors’ behavioral intentions, prompting them to engage in more Prosocial tendencys, such as providing accurate vaccine information to patients and addressing their concerns (49, 50).

Furthermore, prosocial tendency is defined as actions taken with altruistic motivations to benefit others. Doctors with a high sense of social responsibility were more likely to recognize the impact of their actions on patients and society, thus being more motivated to engage in Prosocial tendency. Katz et al. found that healthcare workers’ empathy and altruistic motivations were positively correlated with their Prosocial tendencys, which included providing emotional support and health education to patients (51). Similarly, Burks et al. discovered that medical students’ prosocial values were positively correlated with their Prosocial tendencys, such as volunteering and helping others (52).

The role of Prosocial tendency in reducing vaccine hesitancy had been validated in numerous studies. Opel et al. found that using patient-centered communication strategies, such as encouraging patients to express their concerns and providing personalized advice, can improve their ability to distinguish rumors and their acceptance of vaccines (53, 54). In addition, the relationship between pro social tendencies and vaccine hesitancy may also be influenced by doctors’ own stigmatization. When there is a risk of misunderstanding recommending others to get vaccinated, doctors with high prosocial tendencies may not necessarily consider vaccination as an altruistic behavior. This indicates that we need to comprehensively examine the potential factors that affect vaccine hesitancy (55).

Based on the findings and discussion above, several interventions and policy recommendations can be proposed to mitigate vaccine hesitancy among doctors. First, medical education and training programs should place greater emphasis on cultivating a strong sense of social responsibility among future healthcare professionals. This can be achieved through curricula that highlight the importance of public health, the role of vaccines in disease prevention, and the ethical obligations of doctors to promote the well-being of their patients and society as a whole.

Second, healthcare organizations should provide ongoing training and support to help doctors develop and maintain Prosocial tendencys, such as effective communication skills, empathy, and patient-centered care. This can include workshops, mentorship programs, and opportunities for peer learning and support. By equipping doctors with the skills and resources they need to engage in Prosocial tendencys, healthcare organizations can create a culture that values and promotes these behaviors as an integral part of medical practice.

Finally, policymakers and public health authorities should develop and implement evidence-based strategies to support doctors in addressing vaccine hesitancy among their patients. This can include providing doctors with accurate and up-to-date information about vaccines, developing communication tools and resources to help doctors address common patient concerns, and creating public awareness campaigns to promote the benefits of vaccination. By working collaboratively with healthcare providers and other stakeholders, policymakers can create a supportive environment that empowers doctors to effectively combat vaccine hesitancy.

## 5 Limitation and strength

This study has several limitations that should be considered when interpreting the results. First, the cross-sectional design limits the ability to establish causal relationships between social responsibility, Prosocial tendency, and vaccine hesitancy. Longitudinal studies are needed to further investigate the directionality and temporal relationships between these variables. Second, the reliance on self-reported measures may be subject to social desirability bias, and future research could employ more objective measures to mitigate this bias. Third, the study was conducted among Chinese doctors, which may limit the generalizability of the findings to healthcare professionals in other countries or cultural contexts.

Despite these limitations, this study also has several notable strengths. First, it provides novel insights into the psychosocial factors that may contribute to vaccine hesitancy among healthcare professionals and offers valuable directions for future research and intervention development. Second, the study employed a large and diverse sample of Chinese doctors, enhancing the representativeness of the findings and allowing for the examination of demographic differences in vaccine hesitancy. Third, the use of well-established and validated measures enhances the reliability and validity of the findings and facilitates comparisons with other studies.

Future studies should investigate a range of potential antecedents of vaccine hesitancy among healthcare workers to develop a more comprehensive understanding of this complex phenomenon. Key variables to consider include concerns about vaccine safety, trust in the healthcare system and providers, exposure to misinformation, personal and family health history, cultural and religious beliefs, perceived severity and susceptibility to vaccine-preventable diseases, vaccine-specific factors, organizational policies and norms, psychological characteristics, social network influences, and media consumption habits. By examining these factors, researchers can identify the most salient determinants of vaccine hesitancy in this population and develop targeted, evidence-based interventions to address them. Such efforts will be critical for promoting vaccine acceptance among healthcare workers and, in turn, enhancing public confidence in vaccination programs. Furthermore, future research should employ longitudinal designs and mixed-methods approaches to capture the dynamic nature of vaccine hesitancy and the interplay between individual, social, and contextual factors that shape it over time. By advancing our understanding of vaccine hesitancy among healthcare workers, we can work towards building a more resilient and responsive healthcare workforce in the face of ongoing and emerging public health challenges.

## 6 Conclusion

This study provides valuable insights into the relationship between social responsibility, Prosocial tendency, and vaccine hesitancy among Chinese doctors. The findings suggest that promoting a strong sense of social responsibility and encouraging Prosocial tendencys may be effective strategies for reducing vaccine hesitancy in this population. The negative association between social responsibility and vaccine hesitancy highlights the importance of cultivating a commitment to public health and ethical obligations among healthcare professionals. Furthermore, the partial mediating role of Prosocial tendency underscores the need for interventions that support and facilitate doctor-patient communication, empathy, and patient-centered care. Overall, this study contributes to the growing body of literature on vaccine hesitancy among healthcare professionals and offers valuable recommendations for policymakers, healthcare organizations, and researchers. By prioritizing the development of social responsibility and Prosocial tendencys among doctors, we can work towards creating a healthcare system that is better prepared to address the complex challenges of vaccine hesitancy and promote the health and well-being of individuals and communities.

## Data Availability

The original contributions presented in the study are included in the article/**Supplementary Material**. Further inquiries can be directed to the corresponding author.
